# Pancreaticobiliary fistula associated with intraductal papillary mucinous neoplasm and the simultaneous ampullary carcinoma: A case report

**DOI:** 10.1097/MD.0000000000043563

**Published:** 2025-08-01

**Authors:** Yuki Itagaki, Shoki Sato, Yuko Omori, Hiroyuki Yamamoto, Kotaro Kimura, Hiroki Kushiya, Yusuke Tsunetoshi, Kentaro Kato, Minoru Takada, Yoshiyasu Ambo, Satoshi Ota, Satoshi Hirano

**Affiliations:** a Department of Surgery, Teine Keijinkai Hospital, Sapporo, Japan; b Department of Gastroenterological Surgery II, Faculty of Medicine, Hokkaido University, Sapporo, Japan; c Department of Pathology, Teine Keijinkai Hospital, Sapporo, Japan.

**Keywords:** ampullary adenocarcinoma, intraductal papillary mucinous neoplasm, pancreaticobiliary fistula, pancreatoduodenectomy

## Abstract

**Rationale:**

Intraductal papillary mucinous neoplasms (IPMNs) may perforate into adjacent organs, but pancreaticobiliary fistula caused by IPMN is rare. We present a rare case of pancreaticobiliary fistula due to a branch-duct IPMN, accelerated by a simultaneous ampullary carcinoma.

**Patient concerns:**

A woman in her 70s developed obstructive jaundice and cholangitis after a year of follow-up for IPMN.

**Diagnoses:**

Imaging revealed a branch-duct IPMN with a mural nodule and dilation of the main pancreatic duct, along with a dilated distal bile duct. Perforation into the bile duct and lymphadenopathy were observed. Histopathology later confirmed a high-grade intestinal-type IPMN and an incidental ampullary ductal carcinoma.

**Interventions:**

The patient underwent endoscopic biliary drainage followed by pancreaticoduodenectomy after improvement of cholangitis.

**Outcomes:**

The postoperative course was favorable. The pancreaticobiliary fistula was attributed to mechanical compression by mucin-producing IPMN, complicated by ampullary carcinoma.

**Lessons:**

This case highlights the importance of recognizing synergistic effects of concurrent periampullary neoplasms. Surgical resection may be curative and necessary to control complex biliary obstruction and infection.

## 1. Introduction

Intraductal papillary mucinous neoplasm (IPMN) is known to have cysts that slowly enlarge over several years.^[1]^ IPMN is one of the preinvasive tumors for pancreatic cancer.^[2]^ According to international evidence-based Kyoto guidelines for the management of IPMN, the indication of surgery is based on cyst size, main pancreatic duct (MPD) size, the existence of enhancing mural nodules, symptoms such as obstructive jaundice or pancreatitis, and the rate of increase in cyst size, which is known as high-risk stigmata and worrisome features.^[3]^

IPMN rarely perforates into adjacent organs such as the duodenum, stomach, and bile duct. When it perforates, the mechanism is either mechanical compressive perforation caused by a dilated duct or invasive perforation resulting from carcinoma infiltration with the distraction of stroma.^[4]^ Pancreaticobiliary fistula is a rare complication of IPMN, presenting with obstructive jaundice.^[5]^ According to the reported cases, pancreaticobiliary fistula formation caused by IPMNs is known to take a significant amount of time, often spanning a decade or more.^[6,7]^ Therefore, pancreaticobiliary fistula formation in a year is a rare occurrence. Herein, we report a case of pancreaticobiliary fistulae resulting from mechanical compressive perforation observed during the surveillance of a branch-duct type IPMN (BD-IPMN), co-existing with a nonexposed protruded ampullary ductal carcinoma.

## 2. Case

**Figure 1.**
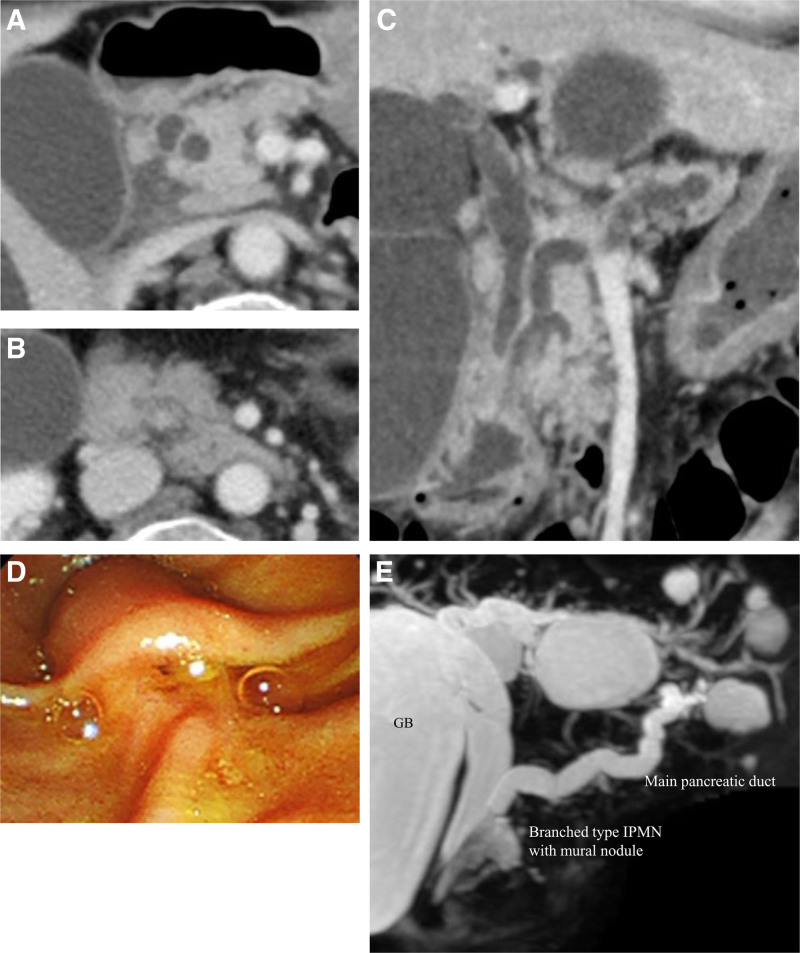
Images at initial diagnosis of intraductal papillary mucinous neoplasm (IPMN). (A) Computed tomography scan image. Axial image of the dilated common bile duct. (B) Axial image of the branch duct-type IPMN and the enhanced mural nodule of the pancreatic head. (C) Coronal image of the dilated main pancreatic duct and common bile duct. The common bile duct wall was thickened. (D) The endoscopic image of the papilla of Vater. (E). Followed up magnetic resonance cholangiopancreatography image revealed a dilated main pancreatic duct with a branch duct-type IPMN and a mural nodule. 12 months after initial diagnosis.

The patient is a female in her 70s. During the workup for gallstone-induced pancreatitis at the previous hospital, contrast-enhanced computed tomography identified a BD-IPMN in the pancreatic head, featuring an 8-mm contrast-enhanced mural nodule and dilation of the MPD. Additionally, the dilated distal common bile duct (CBD) with wall thickening, 9 mm in diameter, was observed (Fig. 1A–C). The endoscopic retrograde cholangiopancreatography did not reveal any abnormalities in the ampulla of Vater (Fig. 1D). The pancreatic juice cytology was class III, indicating an atypical glandular lesion. The patient was diagnosed with an IPMN exhibiting high-risk stigmata, and pancreatoduodenectomy was recommended. However, the patient opted against surgical intervention at that time. The follow-up magnetic resonance cholangiopancreatography showed gradual dilatation of the MPD, increasing from 7 to 9 mm over a 12-month period (Fig. 1E).

**Figure 2.**
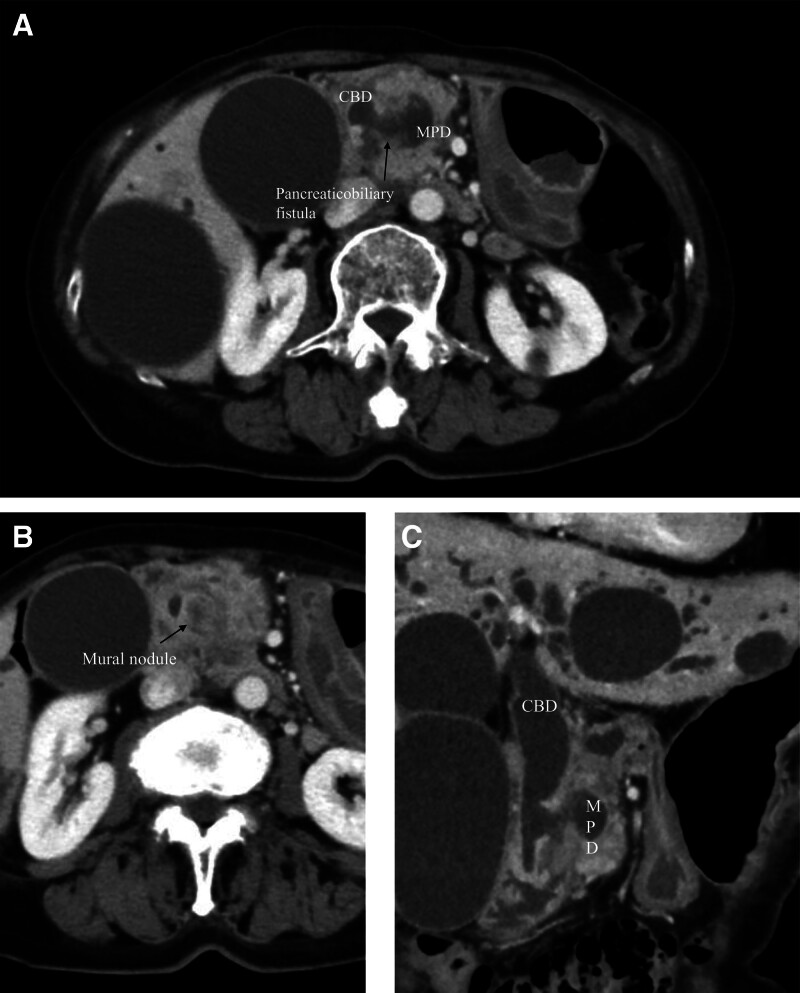
Images of the occurrence of pancreatobiliary fistula 13 months after initial diagnosis. (A) Axial image of pancreaticobiliary fistula. (B) Axial image of mural nodule (arrows). (C) Coronal image of pancreaticobiliary fistula.

After 13 months, she developed obstructive jaundice and acute cholangitis. A Contrast-enhanced computed tomography revealed perforation of the IPMN into the CBD, along with regional and paraaortic lymph node enlargement (Fig. 2A–C). The biliary metallic stent was replaced 3 times, and the plastic pigtail stent was placed once. Despite these interventions, jaundice and cholangitis remained uncontrolled due to recurrent stent occlusions caused by abundant mucus discharge over 5 weeks. Therefore, she was referred to our hospital for further treatment.

The laboratory data on admission showed marked elevation of the hepatobiliary tract enzymes and serum CA19-9 level (Table 1). Following the placement of an endoscopic nasobiliary drainage tube, jaundice and cholangitis gradually improved over 2 weeks. The contrast-enhanced mural nodule, enlarged to 12 mm in height, and obstructive jaundice correspond to high-risk stigmata.^[3]^ Furthermore, the increased serum level of CA19-9, dilation of MPD of 9 mm in diameter, and cystic growth rate of 8 mm per year correspond to worrisome features.^[3]^ The clinical diagnosis was noninvasive intraductal papillary mucinous carcinoma with mechanical perforation or invasive intraductal papillary mucinous carcinoma with invasive perforation. Pancreatoduodenectomy was planned for controlling cholangitis and also for the curative resection of IPMN. If unresectable factors were found during operation, choledochojejunostomy would be performed.

**Table 1:** Laboratory examination on admission.

*Hematology*		
White Blood Cell	8680	/μL
Hemoglobin	11.5	g/dL
Platelet	272	×10^3^/μL
*Coagulation*		
PT	62.8	%
APTT	34.5	sec
Fibrinogen	455	mg/dL
*Serum chemistry*		
Albumin	2.3	g/dL
Urea nitrogen	11.6	mg/dL
Creatinine	0.48	mg/dL
Na	130	mEq/L
K	4.3	mEq/L
Total Bilirubin	3.4	mg/dL
Direct Bilirubin	2.8	mg/dL
Alkaline phosphatase	455	IU/mL
AST	103	IU/mL
ALT	64	IU/mL
γ-GTP	482	U/L
Amylase	255	IU/L
Lipase	9	IU/L
*Tumor marker*		
CEA	2.1	ng/mL
CA19-9	13,233.1	U/mL

**Figure 3.**
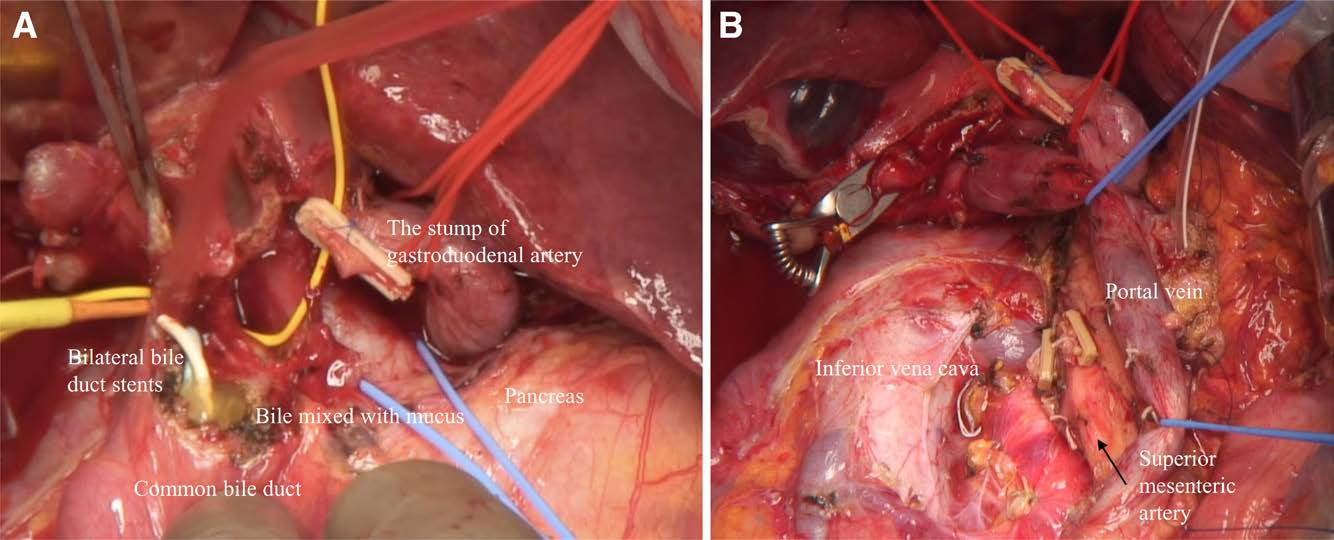
The surgical findings at the pancreaticoduodenectomy. (A) Before resection. The bile mixed with mucus was detected within the common bile duct. (B) After resection. The pancreas was resected at the left edge of the superior mesenteric vein, characterized by a “hard pancreas”.

Intraoperatively, tumor dissemination or metastasis to the para-aortic lymph node was not detected, so a pancreatoduodenectomy was performed (Fig. 3A, B). After resection of the CBD, we identified bile mixed with mucus. The postoperative course was favorable, and the patient was discharged on the 19th postoperative day.

**Figure 4.**
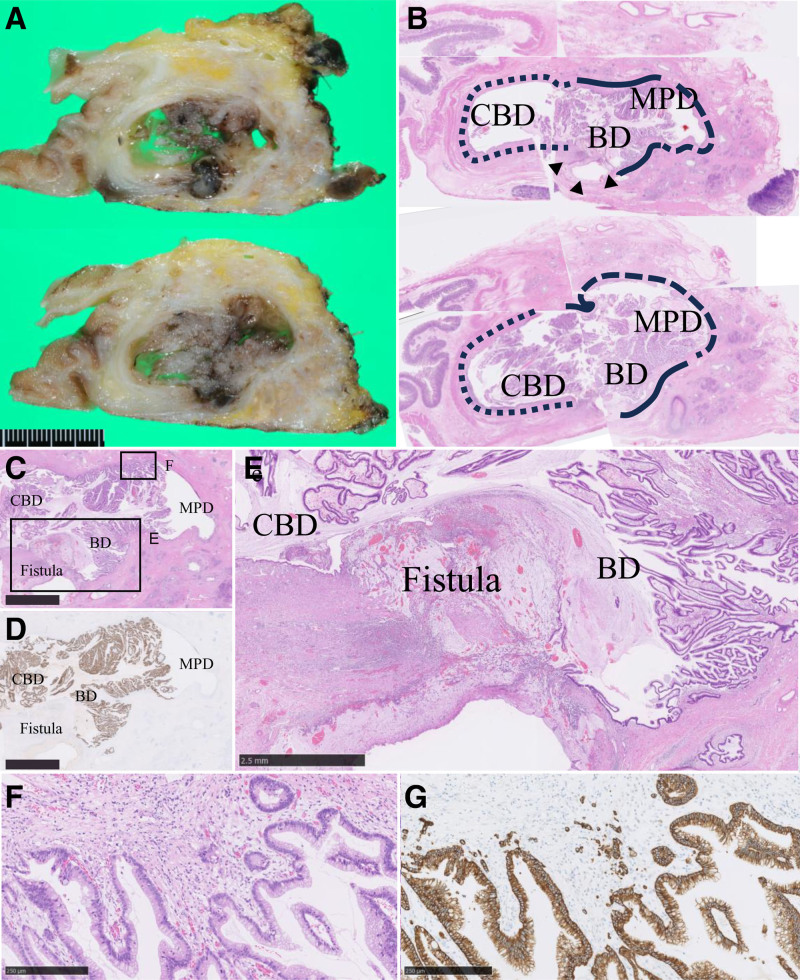
The pathological findings of the intraductal papillary mucinous neoplasm and pancreaticobiliary fistula. (A) Gross findings of the pancreaticobiliary fistula connecting the dilated branch duct of the pancreatic head to the common bile duct. (B) Loupe views of hematoxylin-eosin (HE) stain of the pancreaticobiliary fistula. Dotted lines indicate the common bile duct (CBD), solid lines indicate the dilated branch duct (BD), broken lines indicate the main pancreatic duct (MPD), and arrowheads highlight the acellular mucin pool at the fistula. (C) Low-power view of HE. Papillary growth of the intestinal intraductal papillary mucinous neoplasm (IPMN) with a high-grade dysplasia component in the dilated branch duct. Scale: 5 mm. (D) Low-power view of MUC2 immunostaining. Scale: 5 mm. (E) Granulation with mucin leakage in the surrounding stroma of the fistula. No evidence of tumor invasion was detected in this area. Scale: 2.5 mm. (F) Minimally invasive, poorly differentiated adenocarcinoma, derived from IPMN. Scale: 250 μm. (G) Cytokeratin AE1/AE3 immunostaining. Scale: 250 μm.

Macroscopic examination revealed cystic dilation of the branch duct in the pancreatic head, with mucin accumulation and papillary nodules (Fig. 4A, B). The lesion had directly penetrated the dilated CBD. Additionally, the MPD exhibited diffuse dilation with mucin accumulation.

Histologically, a high-grade IPMN of intestinal type, exhibiting MUC2 positivity, was confined to the cystically dilated branch duct (Fig. 4C, D). Minimal stromal invasion was observed (Fig. 4F, G). At the area of perforation into the CBD, granulation with mucin leakage was noted in the surrounding stroma, but no evidence of tumor invasion was detected (Fig. 4B, E). Although mucin retention was prominent within the MPD, IPMN was not involved. These findings indicated a branch-duct type intestinal IPMN with an associated invasive carcinoma; Ph, tumor size 30 × 20 × 25 mm, invasive size 0.1 mm, pT1a, pN0, cM0, pStage ⅠA according to the Union for International Cancer Control 8th edition,^[8]^ which had mechanically perforated into the CBD.

**Figure 5.**
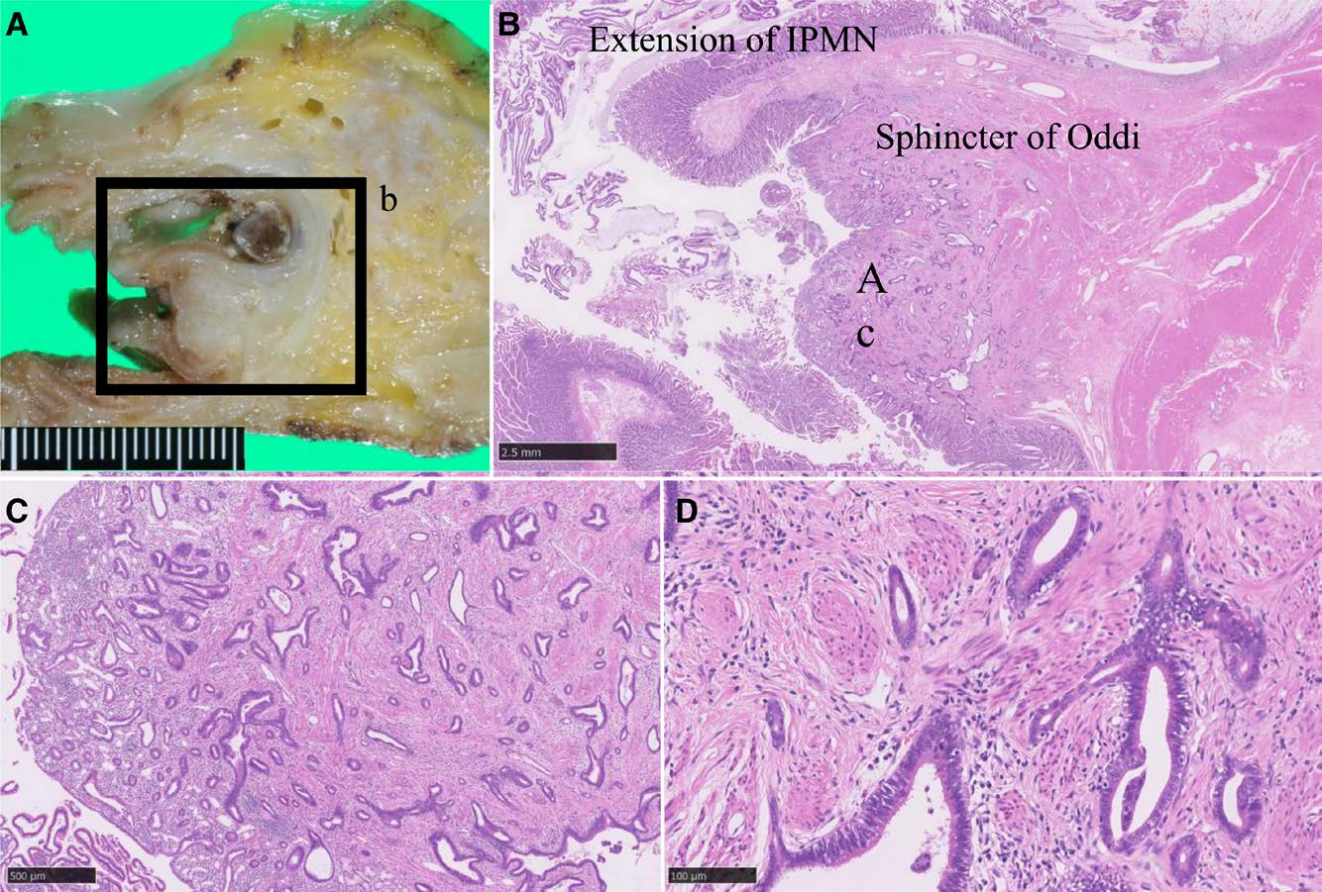
The pathological findings of the ampullary carcinoma. (A) Gross finding of the ampullary carcinoma. (B) Loupe view of hematoxylin-eosin stain (HE). Nodular growth of ampullary ductal carcinoma in the common duct of the ampulla (Ac). Scale: 2.5 mm. (C) Low-power view of HE. Tubule-forming well-differentiated adenocarcinoma infiltrated the sphincter of Oddi. Scale: 500 μm. (D) High-power view of HE. Scale: 100 μm.

Furthermore, an unexpected ampullary carcinoma was identified. This lesion was a nonexposed protruded-type tumor originating from the common duct and ampullary bile duct (Fig. 5A). Histologically, it was a well-differentiated tubular adenocarcinoma with desmoplastic stromal reaction, infiltrating the sphincter of Oddi (Fig. 5B–D). This tumor was diagnosed as an ampullary ductal carcinoma of the pancreatobiliary phenotype, demonstrating MUC1 positivity and MUC2 negativity on immunohistochemical analysis.^[8]^ It was classified as a nonexposed, protruded-type lesion measuring 10 × 8 × 20 mm, and histologically characterized as a well-differentiated tubular adenocarcinoma. According to the TNM classification of the Union for International Cancer Control 8th edition, the tumor was staged as pT1a (denoting invasion into the sphincter of Oddi), pN0, cM0, corresponding to Stage IA.^[8]^

## 3. Discussion

Herein, we present a case of pancreaticobiliary fistula associated with IPMN and the simultaneous existence of ampullary carcinoma. The carcinoma obstructed mucin drainage from the ampulla, leading to excessive mucin accumulation within the bile duct. This resulted in increased intraluminal pressure, accelerating pancreaticobiliary perforation. Unlike typical cases where mucin can partially escape through the ampulla, in this case, a considerable amount of the mucin was directed into the bile duct, making cholangitis control significantly more challenging.

The pancreaticobiliary fistula was diagnosed after 1 year of observation. A previous Japanese journal reported that the frequency of IPMN perforation into other organs ranges from 7.9% to 15.0%, and the perforation into the CBD accounts for 63.5% of these cases, and obstructive jaundice is observed in 97.1%.^[5]^ Treatment for pancreaticobiliary fistula is endoscopic intervention or surgery, such as pancreatoduodenectomy^[6,9–12]^ and choledochojejunostomy as a palliative surgery.^[4,13]^ Endoscopic ultrasound-guided hepatic gastrostomy to control jaundice and cholangitis was also reported.^[7]^

The mechanisms of pancreaticobiliary fistulae associated with IPMNs are mechanical compressive perforation caused by mucus retention in the pancreatic duct and elevation of intraluminal pressure, and perforation due to carcinoma invasion.^[10]^ In this case, granulation in the stromal tissue without invasive cancer cells and mucus leaking into the bile duct was observed. Therefore, the mechanical compression – the increase in intraluminal pressure due to tumor proliferation and mucin accumulation – was considered the cause of the perforation.

IPMNs are known to have a wide spectrum of histological variations associated with clinicopathological characteristics.^[4]^ There are 3 epithelial types as follows: gastric, intestinal, and pancreatobiliary.^[14]^ Intestinal-type IPMNs produce abundant mucin and typically involve MPD, with less invasiveness and a favorable prognosis.^[14]^ Interestingly, the intestinal-type of IPMN in this patient, which was distributed in the branch duct and showed relatively rapid progression, ultimately resulted in a pancreaticobiliary fistula. A previous case report described an intestinal-type IPMN that formed a pancreatobiliary fistula.^[4]^ However, it remains unclear whether the intestinal phenotype itself is a risk factor for fistula formation in IPMNs.

We searched the reported cases of pancreaticobiliary fistula associated with IPMN by MEDLINE via PubMed by MeSH terms, title, and abstract (see Supplemental 1, Supplemental Digital Content, https://links.lww.com/MD/P526). The total of 19 reports of 22 cases and the present case are summarized in Table 2.^[4,6,7,9–12,15–26]^ In 14 cases (14/23, 61%), pancreaticobiliary fistula was evident at the initial diagnosis of IPMN. In 2 cases, fistula was diagnosed several months after the onset of jaundice or in the examination of recurrent pancreatitis. In 6 cases (26%), the interval between the diagnosis of IPMN and the occurrence of pancreaticobiliary fistula formation ranged from 10 to 120 months^[6,7,15,18,24]^ (Table 2). Half of the cases took over 60 months. The shortest time interval was 10 months reported by Corguille et al, which initially revealed the marked dilation of MPD and pseudocyst of the pancreatic head without any surgical intervention.^[15]^

**Table 2:** Summary of case reports of IPMN associated with pancreatobiliary fistula.

Author	Year	Age/sex	Period from IPMN diagnosis to perforation	Initial imaging findings of IPMN	Treatment	Pathological mechanism
Kurihara et al^[25]^	2000	74 M	At the initial consultation		PD	Mechanical perforation
Kurihara et al^[25]^	2000	87 M	Diagnosed 12 months after the first episode of cholangitis		PD	Mechanical perforation
Corguillé et al^[15]^	2002	81 M	10 months after the diagnosis of IPMN	Diffuse dilation of the MPD throughout the gland, with no visible mass lesion; pseudo‑cystic dilation of secondary ducts mainly in the head	Endoscopic metal‑stent placement	NA
Sano et al^[16]^	2003	70 M	At the initial consultation		PD	Mechanical perforation
Okada et al^[11]^	2008	67 M	Diagnosed through postoperative pathology		PD	Mechanical perforation
Nagano et al^[17]^	2009	71 M	At the initial consultation		PD	Invasion of cancer derived from IPMN
Bong et al^[10]^	2011	36 M	At the initial consultation		PD	Invasion of cancer derived from IPMN
Sung et al^[13]^	2011	69 M	At the initial consultation		Endoscopic treatment	Mechanical perforation
Goto et al^[18]^	2012	75 M	18 months after the diagnosis of IPMN	Markedly dilated MPD (45 mm); papillary tumor (20 mm) protruding into the MPD of the distal pancreas and splenic‑vein obstruction	Endoscopic treatment	Mechanical perforation
Nishie et al^[19]^	2013	60s M	At the initial consultation		PD	Mechanical perforation
Mihara et al^[20]^	2015	77 M	At the initial consultation		PD	Mechanical perforation
Mihara et al^[20]^	2015	84 M	At the initial consultation			NA (died before surgery)
Koizumi et al^[12]^	2016	87 F	At the initial consultation			Died of disease
Koizumi et al^[12]^	2016	90 M	At the initial consultation			Died of disease (after metallic‑stent placement)
Komo et al^[21]^	2018	79 M	At the initial consultation		PD	Invasion of cancer derived from IPMN
Ren et al^[4]^	2019	52 M	Diagnosed 4 months after jaundice onset		PD	Mechanical perforation
Khneizer^[22]^	2019	57 M	Diagnosed during evaluation for recurrent pancreatitis		Pancreatic‑duct stent; surgery abandoned due to adhesions	NA
Okamoto et al^[6]^	2019	87 M	120 months after the diagnosis of IPMN	Branch‑duct IPMN gradually progressing to mixed type; MPD > 5 mm and cysts > 5 mm	Endoscopic septotomy of the MPD	NA
Kumar et al^[23]^	2021	60 F	Diagnosed 9 months after jaundice onset		PTBD	NA
Mie et al^[7]^	2021	87 M	108 months after the diagnosis of IPMN	Incidentally noted at distal pancreatectomy; MPD in the residual head gradually enlarged	EUS‑guided hepaticogastrostomy	NA
Patel et al^[24]^	2023	89 M	60 months after the diagnosis of IPMN	Ectatic cystic prominence and focal cystic lesion in proximal pancreas; marked dilatation of proximal MPD	Endoscopic metal‑stent placement	NA
Goncalves et al^[26]^	2024	81 M	At the initial consultation		PD	NA
Present case	2024	70s F	13 months after the diagnosis of IPMN	Branch‑duct IPMN in the pancreatic head with contrast‑enhanced mural nodule; dilation of the MPD and biliary duct	PD	Mechanical perforation

EUS = endoscopic ultrasonography, IPMN = intraductal papillary mucinous neoplasm, MPD = main pancreatic duct, NA = not available, PD = pancreaticoduodenectomy, PTBD = percutaneous transhepatic biliary drainage.

**Figure 6.**
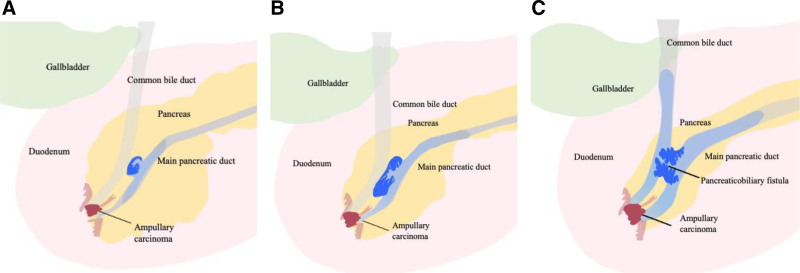
The hypothetical schema for the formation of pancreaticobiliary fistula in the present case. (A) An ampullary ductal carcinoma in the common duct of the ampulla and a branch duct-type intraductal papillary mucinous neoplasm (IPMN) of intestinal-type with high mucus production (blue tumor) developed simultaneously. (B) The ampullary carcinoma interfered with the drainage of mucin-containing pancreatic juice and bile, leading to a synergistic increase in the intraluminal pressure of the pancreaticobiliary system. (C) The progressively dilated branch duct of the pancreas and adjacent bile duct formed a mechanical pancreaticobiliary fistula in a relatively short period.

The present case resulted in perforation within a relatively short period of 13 months. The unique features of the case explain this phenomenon: the simultaneous existence of BD-IPMN of intestinal-type with high mucus production and the ampullary carcinoma. The intraductal papillary neoplasm grew with increased mucin production, progressively dilating the branch duct and spilling mucus, obstructing the MPD. Ampullary carcinoma located in the common and bile ampullary duct further interferes with the drainage of mucin-containing pancreatic juice, leading to increased intraluminal pressure. The cystically dilated branch duct with IPMN eventually perforated into the adjacent CBD. Following the formation of a pancreaticobiliary fistula, a substantial amount of the mucin was directed into the bile duct due to ampullary obstruction, unlike typical perforation causes, where some mucin can drain through the papilla. This resulting semi-closed loop of the pancreatobiliary system, due to the ampullary carcinoma and excessive mucin accumulation in the bile duct, led to uncontrollable cholangitis. The interaction between the 2 neoplasms contributed to the obstruction of the pancreatic and bile ducts, elevated intraluminal pressure, accelerated fistula formation, and resulted in uncontrollable cholangitis. Finally, the synergistic intraductal pressure in both the pancreatic and bile ducts formed pancreaticobiliary fistulae at a rapid phase (Fig. 6A–C).

As there were no factors indicating unresectability, a pancreaticoduodenectomy was performed as a radical resection, resulting in the curative treatment of IPMN and resolution of cholangitis. However, had the ampullary carcinoma been left untreated by selecting choledochojejunostomy, there would have been a potential risk of bile duct stump failure due to the high intraluminal pressure in the closed pancreatobiliary system, compounded by sustained mucin production from the IPMN. Therefore, if the patient’s tolerance to the surgical procedure is not compromised, radical surgical resection should be considered a viable option.

## 4. Conclusion

We have presented a case of rapid formation of pancreaticobiliary fistulae associated with IPMN and ampullary carcinoma. The highly mucin-producing intestinal-type IPMN, along with the ampullary carcinoma, contributed to an accelerated fistula formation due to increased intraluminal pressure, ultimately leading to uncontrollable cholangitis. Notably, the pancreatoduodenectomy was essential not only for managing the IPMN but also for resolving the jaundice and cholangitis in this complex case.

## Author contributions

**Conceptualization:** Yuki Itagaki, Yuko Omori, Hiroyuki Yamamoto, Minoru Takada.

**Data curation:** Yuki Itagaki.

**Project administration:** Yuki Itagaki.

**Supervision:** Shoki Sato, Yuko Omori, Hiroyuki Yamamoto, Kotaro Kimura, Yusuke Tsunetoshi, Kentaro Kato, Minoru Takada, Yoshiyasu Ambo, Satoshi Ota, Satoshi Hirano.

**Visualization:** Hiroki Kushiya.

**Writing – original draft:** Yuki Itagaki, Shoki Sato, Yuko Omori, Minoru Takada, Yoshiyasu Ambo, Satoshi Hirano.

**Writing – review & editing:** Yuki Itagaki, Shoki Sato, Yuko Omori, Hiroyuki Yamamoto, Kotaro Kimura, Hiroki Kushiya, Kentaro Kato, Minoru Takada, Yoshiyasu Ambo, Satoshi Hirano.

## Supplementary Material


