# Supplementary material for: Pancreaticobiliary fistula associated with intraductal papillary mucinous neoplasm and the simultaneous ampullary carcinoma: A case report
Source: Medicine (Baltimore). 2025 Aug 1;104(31):e43563. doi: 10.1097/MD.0000000000043563 (PMC12323950; doi:10.1097/MD.0000000000043563)
Supplement: Supplementary file 1 [file medi-104-e43563-s001.docx]

**Supplement 1.**

#1 **Intraductal Papillary Mucinous Neoplasms [MeSH Terms]**

#2 IPMN [Title/Abstract]

#3 Biliary Fistula [MeSH Terms]

#4 Pancreaticobiliary fistula [Title/Abstract]

#5 pancreatobiliary fistula [Title/Abstract]

#6 #1 OR #2

#7 #3 OR 4 OR #5

#8 #6 AND #7

20 Results
